# Supplementary material for: Tactile emoticons: Conveying social emotions and intentions with manual and robotic tactile feedback during social media communications
Source: PLoS One. 2024 Jun 12;19(6):e0304417. doi: 10.1371/journal.pone.0304417 (PMC11168615; doi:10.1371/journal.pone.0304417)
Supplement: S1 File — (DOCX) [file pone.0304417.s001.docx]

Supplementary Material for:

Tactile Emoticons: Conveying Social Emotions and Intentions with Manual and Robotic Tactile Feedback During Social Media Communications

Alkistis Saramandi^1¶*#a^, Yee Ki Au^1¶^, Athanasios Koukoutsakis^1^, Caroline Yan Zheng^2,3^, Adrian Godwin, Nadia Bianchi-Berthouze^4^, Carey Jewitt^5^, Paul M Jenkinson^1,6^ & Aikaterini Fotopoulou^1^

^1^Department of Clinical, Educational and Health Psychology, University College London, London, United Kingdom

^2^Royal College of Art, London, United Kingdom

^3^KTH Royal Institute of Technology, Stockholm, Sweden

^4^UCL Interaction Centre, University College London, London, United Kingdom

^5^UCL Knowledge Lab, Culture Communication and Media, University College London, London, United Kingdom

^6^Faculty of Psychology, Counselling and Psychotherapy, The Cairnmillar Institute, Melbourne, Australia

^#a^Current Address: Department of Clinical, Educational and Health Psychology, University College London, London, United Kingdom

^*^ Corresponding Author:

E-mail: [alkistis.saramandi.15@ucl.ac.uk](mailto:alkistis.saramandi.15@ucl.ac.uk) (AS)

^¶^These authors have shared the first authorship.

keywords: *affective touch, emoticons, mediated touch, digital communication, skin conductance rate, heart rate variability, regulation*

**Supplementary Material**

In this section we provide further details on the Methods of our two experiments, specifically a table with all the pre-constructed posts used in our two studies, details on our power analyses, our additional measures used in our secondary and exploratory analyses, as well as the respective results.

**Supplementary Methods: Experiments 1 and 2**

Table S1 includes the pre-constructed posts used across the two experiments. Posts marked with an asterisk (*) were not included in Experiment 2. Posts 1-32 are of positive valence and posts 33-64 are of negative valence.

| **Table S1. Pre-constructed posts for Experiments 1 and 2.** | |
| --- | --- |
| **Number** | **Post** |
| 1 | Celebrating five years of friendship |
| 2 | Delighted my brother is moving back to town |
| 3 | Delighted that our local council decided to provide shelter to all homeless people in the area |
| 4 | Got VIP invitation by a friend to join him at his club for an exciting event |
| **5*** | Happy and relieved I passed my French exam |
| 6 | Helped my mom fold the clean bedsheets - they smell so fresh! |
| 7 | I am getting married |
| 8 | I completed a marathon to support my sister's Charity |
| 9 | I got a new job |
| 10 | I got a promotion |
| 11 | I graduated |
| **12*** | I had a delicious smoothie |
| 13 | I just discovered an amazing song |
| 14 | I just had the longest catch up with my best friend |
| 15 | I was delighted with the new tax rule |
| 16 | I was upgraded to first class |
| 17 | Joined new Charity and loving the new friends I am making |
| 18 | Just added picture of my birthday party looking glam [imagine that you see a picture of that individual] |
| 19 | Just managed to help my friend negotiate a better rent for her flat. Oh, the pride |
| **20*** | Just tried my new dress and my mirror says it looks great |
| **21*** | Looking forward to enjoying a family holiday at home at the end of this very busy week |
| 22 | Managed to learn new software that made me really fast and efficient at work |
| 23 | Met the nicest lady at the bus stop today who lent me spare change |
| 24 | My best friend threw a surprise party for me |
| 25 | My boss told me my work has been steadily improving |
| **26*** | My friend gave me a nice haircut today |
| 27 | My friend got me a ticket to my favourite band's concert |
| **28*** | My package finally arrived today and the shoes fit so well |
| 29 | New neighbours moved in and invited us in lovely housewarming party |
| 30 | New physio sorted out my back pain and I started exercising again |
| **31*** | Re-decorated my living room and it looks amazing |
| **32*** | Signed up at the local shelter with my siblings to foster kittens |
| 33 | A lady cut into the queue in front of me at the busy canteen today |
| 34 | Annoyed as I have to wait two weeks to see my GP despite severe pain in my neck |
| **35*** | Appalled by the council's response to the victims of floods |
| 36 | Bouncer spoiled the night by not letting in a friend I was waiting in a club yesterday |
| 37 | Broke my leg before an important sports fundraiser for my cousin's foundation |
| 38 | Computer crashed at work and lost one month's worth of data |
| 39 | Failed my driving test |
| 40 | Feeling a bit apprehensive about having to move abroad by myself next month |
| **41*** | Got scammed by an online shop and lost my money |
| 42 | Had fight with best friend |
| 43 | Had to say goodbye to my favourite colleague today |
| 44 | I am sleep deprived and irritated with our new, loud and inconsiderate neighbours |
| 45 | I am very sad about the recent elections |
| **46*** | I ate a disgusting food |
| 47 | I failed an exam |
| 48 | I got told off for something that was not my fault |
| **49*** | I have a leak in our living room and new sofa is destroyed |
| 50 | I made a mistake at work |
| 51 | I missed my flight to holiday with friends |
| 52 | I mixed up recipients and just sent my boss an embarrassing text. Oh, the shame |
| **53*** | I slipped on mud and my white jeans are now dirty |
| 54 | I think my mother forgot my birthday |
| 55 | I was dumped |
| 56 | I was made redundant |
| 57 | I watched a horrible movie |
| 58 | Just added a picture of me, drunk and on the floor last Friday night [imagine that you see a picture of that individual] |
| **59*** | Lost the headphones my friend let me borrow |
| **60*** | My close friend didn’t turn up at my first piano recital today |
| 61 | My flight back from Germany was cancelled |
| 62 | Not feeling confident with my looks this morning |
| **63*** | Sad the dinner party with my friends next week got cancelled |
| 64 | Shook by the sudden death of our beloved dog |

**Supplementary Methods: Experiment 1**

**Participants**

An *a priori* power analysis was conducted using G*Power , to determine the number of participants needed to achieve the smallest effect size of interest [1]. We used the G*Power conventions for a medium effect size (*f*=0.25, power (1-β) = 0.95, significance level (α)=0.05), based on Cohen’s suggestions [2], [3]. Also, given the recognised difficulties in conducting power analysis for multilevel modelling analyses and lack of standardised procedures (4,5), we based our power analysis on a standard repeated measures, within subjects ANOVA, given that it would give us an estimate of the required sample size to detect the main effects of interest (i.e., feedback mode). This calculation yielded a minimum required sample of N=36 participants.

**Secondary Measures**

We were also interested in examining whether the number of ‘likes’ participants thought each post should receive (as measured before the main task, and hereafter referred to as pre-task likes), would influence the amount of perceived social intent during the main task. Participants were shown one post at a time and asked to report how many ‘likes’ they thought it should get on a scale of 0 (*not at all*) to 100 (*maximum ‘likes’*).

The main dependent variable (DV) was the amount of ‘social approval and support’ participants rated after each feedback trial (hereafter referred to as ‘social intent’) on a 0 (*no approval and support at all*) to 100 (*extreme approval and support*) scale. Additionally, to examine whether our different feedback modes would have any implicit effects on perceived social intent, exploratory analyses were run on a secondary DV, which was the expected number of ‘likes’ participants thought each ‘post’ would receive, measured after the main task (hereafter referred to as post-task likes).

**Psychometric Measures**

We used self-report questionnaires to examine whether traits of anxiety, attitudes towards physical touch, alexithymia and social media influenced participants’ perceived social intent during the main task.

Social Anxiety Questionnaire (SAQ-30; [6]) : The 30-item self-report questionnaire is designed to measure specific and/or generalised social phobia/anxiety in adults from general and clinical populations. The SAQ-30 consists of five factors: (i) speaking in public/talking with people in authority, (ii) interaction with the opposite sex, (iii) assertive expression of annoyance, disgust, or displeasure, (iv) criticism and embarrassment, and (v) interaction with strangers. Each factor includes six items, each of which is rated on a 1 (*not at all* *or very slightly)* to 5 (*very high or extremely high*) point Likert scale. The SAQ-30 is a valid and reliable self-report measure of social anxiety with Cronbach’s α ranging from .811 to .922 in the general population. In Experiment 1 the internal consistency was excellent with Cronbach’s α=.978.

Social Touch Questionnaire (STQ; (7)): The STQ is used to study attitudes towards physical touch on a 0 (*not at all*) to 4 (*extremely*) point Likert scale. For instance, it is used to measure attitudes towards giving vs. receiving touch, touch involving an acquaintance vs. a stranger, touch occurring in a public vs. private place, or touch having sexual vs. non-sexual connotations [7]. The internal consistency is excellent in general populations (Cronbach’s α=.88; [8]). Our sample also showed optimal consistency (Cronbach’s α=.744).

Body Awareness Questionnaire (BAQ; [9]): The BAQ is an 18-item questionnaire which measures attentiveness to normal non-emotive body processes, and in particular sensitivity to body cycles and rhythms on a 1 (*not at all true about me*) to 7 (*very true about me*) scale, and higher scores indicating greater body awareness. The BAQ consists of four factors, namely (i) note of responses to changes in body process, (ii) predict bodily sensation, (iii) sleep-wake cycle, and (iv) onset of illness. The questionnaire has shown to be internally consistent in an undergraduate sample (Cronbach’s α=.82; [9]). Our sample also demonstrated excellent internal consistency (Cronbach’s α=.89).

Toronto Alexithymia Scale (TAS-20; [10]): The 20-item TAS is a validated and reliable self-report measure of alexithymia in both clinical and general populations, assessing individual difficulty of identifying and describing feelings, and externally oriented thinking on a 1 (*strongly disagree*) to 5 (*strongly agree*) Likert scale. Individual scores may range from 20 to 100, with a total score above 61 being the cut-off score for alexithymia according to general population studies ([11], [12]. The scale has demonstrated good internal consistency (Cronbach’s α = .86; [13]), and internal consistency was also optimal in our sample (Cronbach’s α=.744).

Social Networking Site survey (SNS; [14]): The SNS was used to gage individual variability in habits and relationship with social media use. The self-report measure includes two subscales – the SNS Featured Usage and the SNS Affective Experiences subscales – allowing to examine featured usage (e.g., updating one’s status, measured on a scale of 1 (*never*) to 7 (*multiple times a day*)) as well as the frequency of pleasant (e.g., happiness, joy) and unpleasant (e.g., anxiety, anger) affects (measured on a scale of 1 (*never*) to 7 (*always*); [14]). The internal consistency of the two subscales is excellent (Cronbach’s α=.90 and .85, respectively; [14]). The internal consistency in our sample was good (Cronbach’s α=.831 and .789, respectively).

**Data Analysis**

Specifically, for the second hypothesis we used social intent as the DV, and the number of likes estimated during pre-task as the IV, whilst valence, feedback mode and participant ID were used as random effects. For the third hypothesis, the number of likes estimated during post-task were used as the DV, and social intent was used as IV (valence, feedback mode and participant ID as random effects). Finally, we ran correlations between social intent and the psychometric measures (SAQ-30, BAQ, STQ, TAS-20, and SNS), to explore how these traits and social media usage was associated with the perception of feedback in a simulated social media environment.

**Supplementary Results: Experiment 1**

**Hypothesis 2: Participants who expected posts to receive more ‘likes’ also perceived greater ‘social approval and support’ in the main task**

A significant main effect of “pre-task likes”, indicated that participants who expected posts to receive more likes (as measured during the pre-task section) also reported greater social intent during the main task (*β*=0.11, SE=0.02, *t*(5.03), 95% CI=0.07-0.15, *p*<0.001, ICC=0.30, R_marginal_^2^=0.012, R_conditional_^2^=0.312) .

**Hypothesis 3: Participants who perceived higher social intent during the main task, expected to receive more “likes” in the post task**

We found a significant main effect of social intent, indicating greater social intent during the main task was predictive of more “likes” expectations during post-task (*β*=0.13, SE=0.02, *t*(6.83), 95% CI=0.09-0.17, *p*<0.001, ICC=0.37, R_marginal_^2^=0.018, R_conditional_^2^=0.382).

**Questionnaire Analyses**

Correlations were used to explore the effects of individual differences. Using overall questionnaire scores, Pearson’s correlations revealed positive correlations between measurements of (i) social anxiety (SAQ_30) and social touch (STQ; r=.365, *p*=.029) and (ii) SAQ_30 and alexithymia (TAS; r=.477; *p*=.003). While participants’ attitudes towards physical touch and alexithymia remained relevant to their experience of affective touch, social anxiety was not included in further analyses to avoid issues of multicollinearity.

We further investigated correlations between perceived social intent and social touch (STQ), alexithymia (TAS), body awareness (BAQ), and social media use (SNS). We found a positive correlation between social intent and TAS (r=0.038; *p*=-.02), and a negative correlation between social intent and BAQ (r=-0.043, *p*=0.043). The ‘interactive usage’ factor in the SNS – which includes a rating of ‘use social media to update social status’ – was also positively correlated with the overall perceived social intent (r=.088; *p*<.001). That is, the more participants use their social media accounts to post status updates and pictures, the more social intent they perceived following feedback. Other correlations did not yield significant results (*p*>.05).

**Supplementary Methods: Experiment 2**

**Participants**

An *a priori* required sample size calculation was conducted using the same parameters as in Experiment 1 and the effect size (*f*=0.203) from the trend between valence and feedback mode found in Experiment 1. This yielded a required minimum sample size of N=54 participants to achieve a power of 95% (G*Power; [15]).

**Psychometric Measures**

State-Trait Anxiety Inventory (STAI; [16]): We used the trait subscale from the STAI to assess trait anxiety. The STAI is a 40-item self-report measure (20 items for state and 20 for trait), rated on a 4-point likert scale, ranging from 1 (*not at all*) to 4 (*very much*). The internal consistency of the STAI is excellent (Cronbach’s α ranging from .86 to .95; (16)), while our sample’s consistency was acceptable, Cronbach’s α=.62.

#### Rejection Sensitivity Questionnaire for adults (RSQ-A; [17], [18]): The RSQ-A was used to assess rejection sensitivity in our sample. Rejection sensitivity is a cognitive-affective processing disposition to anxiously expect rejection, shaped by cognitive-social learning theory and triggered in situations when either rejection or acceptance is likely. The RSQ-A has great internal consistency (Cronbach’s α=.89) and is used to measure the level of rejection concern and rejection expectancy in several situations (e.g., *You ask your parents or another family member for a loan to help you through a difficult financial time. How concerned or anxious would you be over whether or not your family would want to help you. (1 – very unconcerned to 6 – very concerned); I would expect that they would agree to help as much as they can (1 – very unlikely to 6 – very likely*). Our sample also showed good internal consistency – Cronbach’s α=.79.

#### Experience in Close Relationships – Revised questionnaire (ECR-R; [19]): The ECR-R is a self-report measure to assess attachment anxiety and attachment avoidance and is more reflective of real-life attachment in comparison to other questionnaires that yield results in a categorical scale (ECR-R results are continuous scores). ECR-R has high internal consistency (Cronbach’s α equal to .90 or higher for both the attachment anxiety and avoidance dimensions; [20]–[22]), and participants are asked to indicate how they feel in emotionally intimate relationships and generally how they experience relationships on a scale of 0 (*strongly disagree*) to 4 (*strongly agree*). In our analyses we used the attachment anxiety subscale, which showed great internal consistency (Cronbach’s α=.93).

#### In addition, the STQ mentioned in Experiment 1 was used. It showed good internal consistency in our sample, Cronbach’s α=.80. Questions from the Feature Usage subscale of the SNS questionnaire’s were also used ([14]; also administered in Experiment 1).

**Data Analysis**

We ran an MLM with baseline-corrected HR, SCR and HRV scores as our separate DVs, feedback mode as our IV, participant ID and block as random effects. Furthermore, in separate MLMs we examined the interaction between feedback mode and trait anxiety and repeated this analysis for other traits too, namely rejection sensitivity and attachment anxiety. Irrespective of the two-way interaction effect, the analyses on the interaction between feedback mode and traits were also run separately in each valence.

HR, SCR and HRV scores per participant were calculated as follows:

1. HR: The data obtained from the Empatica E4 interface contain the average heart rate values computed in spans of 10 seconds. The sampling rate is obtained in Hz, and all UNIX timestamp values are 1 second apart.
2. SCR: The output of Empatica E4 includes a column in which data are collected in MicroSiemens (µS) at 4Hz.
3. HRV: We used the inter-beat interval (IBI) output file to calculate the difference in the time elapsed between two successive IBIs to calculate the root mean square (RMSSD) and then transformed the RMSSD values by the natural algorithm (ln) as seen in Rominger et al. [23].

For each of the physiological measures, we obtained six scores: one score for the pre-task baseline measurement, and one for the post-task measurements, and four scores for each one of the main task blocks. The different scores were distinguished using the timestamp information from the respective file we downloaded from the Empatica E4 interface. The HR and SCR scores were baseline-corrected prior to the analyses, i.e., we subtracted the mean HR and SCR scores from each block (e.g., visuotactile feedback mode on negative posts, visual feedback mode on positive posts, etc.) from the initial 5-min baseline HR and SCR scores, respectively.

For control purposes, we also collected baseline ratings of pleasantness (on a scale of 0 (*not at all pleasant*) to 100 (*extremely pleasant*) following touch delivered by the S-CAT and via brush strokes at CT-optimal (6cm/sec) and CT-suboptimal speeds (36cm/sec). We then ran an MLM with touch medium (manual touch via brush strokes vs. S-CAT) and touch velocity (optimal vs. suboptimal CT-touch) as interacting IVs. We examined the main effect and interaction of these IVs on participants’ pleasantness ratings. Participant ID was added as a random effect.

**Supplementary Results: Experiment 2**

**Hypothesis 4a: Heart rate (HR) was not more downregulated in the visuotactile feedback mode as opposed to the visual feedback mode.**

When using HR as our DV, we found no significant main effect of feedback mode (*β*=-0.15, SE=0.33, *t*(-0.47), 95% CI=-0.79-0.48, *p*=0.638, ICC=0.91, R_marginal_^2^=0.000, R_conditional_^2^=0.910), no significant interaction between feedback mode and valence (*β*=0.31, SE=0.65, *t*(0.47), 95% CI=-0.97-1.58, *p*=0.636, ICC=0.91, R_marginal_^2^=0.000, R_conditional_^2^=0.91), and no significant main effect of feedback mode when examining the effect of feedback mode on each valence separately (Positive Valence: (*β*=-0.00, SE=0.56, *t*(-0.01), 95% CI=-1.10-1.10, *p*=0.998, ICC=0.87, R_marginal_^2^=0.000, R_conditional_^2^=0.873); Negative Valence: (*β*=-0.30, SE=0.44, *t*(-0.673), 95% CI=-1.17-0.57, *p*=0.501, R_marginal_^2^=0.005, R_conditional_^2^=NA)). Additionally, we found no significant interaction between feedback mode and trait anxiety, feedback mode and rejection sensitivity, or between feedback mode and attachment anxiety (in all posts, but also within each valence separately; all *p* values >0.05).

**Hypothesis 4b: Skin conductance rate (SCR) was not downregulated more in the visuotactile feedback mode as opposed to the visual feedback mode.**

No significant main effects or interactions were found when using SCR values instead of HR scores (Main effect of feedback mode: (*β*=-0.02, SE=0.03, *t*(-0.91), 95% CI=-0.07-0.03, *p*=0.365, ICC=0.89, R_marginal_^2^=0.001, R_conditional_^2^=0.887); Interaction between feedback mode and valence (*β*=-0.04, SE=0.05, *t*(-0.793), 95% CI=-0.14-0.06, *p*=0.428, ICC=0.89, R_marginal_^2^=0.001, R_conditional_^2^=0.890); Positive Valence: (*β*=-0.05, SE=0.05, *t*(-0.931), 95% CI=-0.16-0.06, *p*=0.990, ICC=0.73, R_marginal_^2^=0.003, R_conditional_^2^=0.727); Negative Valence: (*β*=-0.00, SE=0.02, *t*(-0.268), 95% CI=-0.04-0.03, *p*=0.789, ICC=0.98, R_marginal_^2^=0.000, R_conditional_^2^=0.976)) or when looking at the interaction between feedback and the rejection sensitivity and anxiety traits (all *p* values >0.05). Interestingly, the interaction between visuotactile feedback and rejection sensitivity was significant, when looking at the posts of positive valence, only (*β*=0.01, SE=0.01, *t*(2.12), 95% CI=0.00-0.02, *p*=0.034, ICC=0.89, R_marginal_^2^=0.019, R_conditional_^2^=0.896). Specifically, SCR score variability was better explained by the visuotactile feedback mode as opposed to the visual feedback mode, and the effect of feedback mode on SCR was increased in individuals with higher rejection sensitivity scores.

**Hypothesis 4c: Heart rate variability (HRV) was not downregulated more in the visuotactile feedback mode as opposed to the visual feedback mode.**

When looking at changes in HRV following feedback mode, we found no significant main effects of feedback mode (*β*=0.0008, SE=0.001, *t*(0.769), 95% CI=-0.0012-0.0027, *p*=0.442, ICC=0.93, R_marginal_^2^=0.000, R_conditional_^2^=0.927), interactions of feedback mode with valence (*β*=0.0024, SE=0.002, *t*(1.195), 95% CI=-0.0015-0.0063, *p*=0.232, ICC=0.93, R_marginal_^2^=0.001, R_conditional_^2^=0.926), or within each valence (Positive Valence: (*β*=0.0018, SE=0.0016, *t*(1.148), 95% CI=-0.0013-0.0050, *p*=0.251, ICC=0.90, R_marginal_^2^=0.001, R_conditional_^2^=0.899); Negative Valence: (*β*=-0.0006, SE=0.0014, *t*(-0.408), 95% CI=-0.0034-0.0022, *p*=0.683, ICC=0.93, R_marginal_^2^=0.000, R_conditional_^2^=0.929)). We also found no significant effect of rejection sensitivity or attachment anxiety on HRV (all *p* values >0.05). However, we found a significant interaction between feedback mode and trait anxiety, both across valences and within posts of negative valence only (Across valences: (*β*=0.0005, SE=0.0002, *t*(3.254), 95% CI=0.0002-0.008, *p*=0.001, ICC=0.93, R_marginal_^2^=0.064, R_conditional_^2^=0.933); Negative Valence: (*β*=0.0005, SE=0.0002, *t*(2.082), 95% CI=0.0000-0.001, *p*=0.037, ICC=0.92, R_marginal_^2^=0.065, R_conditional_^2^=0.921)). This result suggests that the visuotactile feedback mode explained more of the HRV variance as compared to the visual feedback mode, and the effect of feedback mode on HRV differences increased as trait anxiety scores increased.

**Control Analysis: Pleasantness ratings were predicted by touch velocity but not by touch medium.**

We examined the main effect of touch medium and touch velocity and the interaction of these two variables on participants’ pleasantness ratings. We found a significant main effect of touch velocity, in that touch delivered at CT-optimal speeds, resulted in higher pleasantness ratings than touch delivered at CT-suboptimal speeds (*β*=20.78, SE=3.03, *t*(6.85), 95% CI=14.83 – 26.73, *p*<0.001, ICC=0.43, R_marginal_^2^=0.194, R_conditional_^2^=0.539). However, the effect of touch medium on pleasantness ratings was not significant (*β*=-2.81, SE=3.05, *t*(3.05), 95% CI=-8.78 – 3.16, *p*=0.356, ICC=0.43, R_marginal_^2^=0.194, R_conditional_^2^=0.539). The, interaction between these two IVs was also not significant (*β*=-3.36, SE=4.29, *t*(-0.78), 95% CI=-11.76 – 5.04, *p*=0.433, ICC=0.43, R_marginal_^2^=0.194, R_conditional_^2^=0.539). This result suggests that participants reported significantly higher pleasantness ratings following touch delivered at CT-optimal speeds (6cm/sec) compared to touch delivered at CT-suboptimal speeds (36cm/sec), irrespective of how the touch was delivered (touch medium).

**References**

1. Lakens D. Sample Size Justification. Collabra Psychol. 2022 Mar 22;8(1):33267.

2. Clever L, Colaianni LA, Davidoff F, Horton R, Kassirer JP, Angell M, et al. eweb:173521. International Committee of Medical Journal Editors; 1997 [cited 2023 Mar 19]. Uniform Requirements for Manuscripts Submitted to Biomedical Journals: Special Report. Available from: https://repository.library.georgetown.edu/handle/10822/901209

3. Kang H. Sample Size Determination and Power Analysis Using the G*Power Software. J Educ Eval Health Prof. 2021;

4. Snijders T. Power and sample size in multilevel modeling. 2005 Jan 1;3:1570–3.

5. Maas CJM, Hox JJ. Sufficient Sample Sizes for Multilevel Modeling | Methodology [Internet]. 2005 [cited 2023 Mar 19]. Available from: https://econtent.hogrefe.com/doi/abs/10.1027/1614-2241.1.3.86

6. Caballo VE, Salazar IC, Irurtia MJ, Arias B, Hofmann SG. The Multidimensional Nature and Multicultural Validity of a New Measure of Social Anxiety: The Social Anxiety Questionnaire for Adults. Behav Ther. 2012 Jun 1;43(2):313–28.

7. Wilhelm FH, Kochar AS, Roth WT, Gross JJ. Social anxiety and response to touch: incongruence between self-evaluative and physiological reactions. Biol Psychol. 2001 Dec 1;58(3):181–202.

8. Trotter PD, McGlone F, Reniers RLEP, Deakin JFW. Construction and Validation of the Touch Experiences and Attitudes Questionnaire (TEAQ): A Self-report Measure to Determine Attitudes Toward and Experiences of Positive Touch. J Nonverbal Behav. 2018 Dec 1;42(4):379–416.

9. Shields SA, Mallory ME, Simon A. The Body Awareness Questionnaire: Reliability and Validity. J Pers Assess. 1989 Dec 1;53(4):802–15.

10. Bagby RM, Parker JDA, Taylor GJ. The twenty-item Toronto Alexithymia scale—I. Item selection and cross-validation of the factor structure. J Psychosom Res. 1994 Jan 1;38(1):23–32.

11. Honkalampi K, Hintikka J, Laukkanen E, Viinamäki JLH. Alexithymia and Depression: A Prospective Study of Patients With Major Depressive Disorder. Psychosomatics. 2001 May 1;42(3):229–34.

12. Franz M, Popp K, Schaefer R, Sitte W, Schneider C, Hardt J, et al. Alexithymia in the German general population. Soc Psychiatry Psychiatr Epidemiol. 2008 Jan 1;43(1):54–62.

13. Preece DA, Becerra R, Allan A, Robinson K, Chen W, Hasking P, et al. Assessing alexithymia: Psychometric properties of the Perth Alexithymia Questionnaire and 20-item Toronto Alexithymia Scale in United States adults. Personal Individ Differ. 2020 Nov 1;166:110138.

14. Shi Y, Luo Y, Yang Z, Liu Y, Cai H. The Development and Validation of the Social Network Sites (SNSs) Usage Questionnaire. 2014. 113 p.

15. Faul F, Erdfelder E, Buchner A, Lang AG. Statistical power analyses using G*Power 3.1: Tests for correlation and regression analyses. Behav Res Methods. 2009 Nov 1;41(4):1149–60.

16. Spielberger CD. State-Trait Anxiety Inventory for Adults. 1983;

17. Berenson KR, Gyurak A, Ayduk Ö, Downey G, Garner MJ, Mogg K, et al. Rejection sensitivity and disruption of attention by social threat cues. J Res Personal. 2009 Dec 1;43(6):1064–72.

18. Downey G, Feldman SI. Implications of rejection sensitivity for intimate relationships. J Pers Soc Psychol. 1996;70:1327–43.

19. Brennan KA, Clark CL, Shaver PR. Self-report measurement of adult attachment: An integrative overview. In: Attachment theory and close relationships. New York, NY, US: The Guilford Press; 1998. p. 46–76.

20. Krahé C, von Mohr M, Gentsch A, Guy L, Vari C, Nolte T, et al. Sensitivity to CT-optimal, Affective Touch Depends on Adult Attachment Style. Sci Rep. 2018 Sep 28;8(1):14544.

21. Ravitz P, Maunder R, Hunter J, Sthankiya B, Lancee W. Adult attachment measures: A 25-year review. J Psychosom Res. 2010 Oct 1;69(4):419–32.

22. Sibley CG, Fischer R, Liu JH. Reliability and Validity of the Revised Experiences in Close Relationships (ECR-R) Self-Report Measure of Adult Romantic Attachment. Pers Soc Psychol Bull. 2005 Nov 1;31(11):1524–36.

23. Rominger C, Weber B, Aldrian A, Berger L, Schwerdtfeger AR. Short-term fasting induced changes in HRV are associated with interoceptive accuracy: Evidence from two independent within-subjects studies. Physiol Behav. 2021 Nov 1;241:113558.
