# Supplementary material for: Tactile emoticons: Conveying social emotions and intentions with manual and robotic tactile feedback during social media communications
Source: PLoS One. 2024 Jun 12;19(6):e0304417. doi: 10.1371/journal.pone.0304417 (PMC11168615; doi:10.1371/journal.pone.0304417)
Supplement: Attachment — Submitted filename: Response to Reviewers.docx [file pone.0304417.s002.docx]

23 April 2024

Dear Dr Bruno,

Thank you for inviting us to submit a revised version of our manuscript entitled, “Tactile Emoticons: Conveying Social Emotions and Intentions with Manual and Robotic Tactile Feedback During Social Media Communications” (PONE-D-23-29955) for consideration in *PlosOne*. We are grateful for the positive comments received by the reviewers regarding our manuscript and have now addressed their questions and comments below and in the manuscript (changes highlighted in yellow). We have also uploaded two versions of our revised Supplementary Material (one with highlighted track changes and one without). The comments made by Reviewer #1 are highlighted in green, and the comments made by Reviewer #2 are highlighted in blue. We would also like to note that the grant information in the ‘Financial Disclosure’ section is the correct information. We were not able to add the UCL Social Science + award information in the Funding Information as the system did not allow us to manually add a funder that was not shown an option in the dropdown menu in that section. We would be grateful if you could please let us know how to fix this.

We hope the paper is now suitable for publication and look forward to hearing from you in due course.

On behalf of all the authors, yours sincerely,

Corresponding author [anonymised in line with journal guidelines]

**Reviewer #1**

Overall, I think this is a very nice study that will make a good contribution to the literature. The experiments, analyses and conclusions are all sound. However, I have some substantial concerns with some aspects of the presentation, which leads me to recommend a major revision.

**Reviewer #1, Major Comment 1:**

The S-CAT appears to be an apparent motion device, with pneumatic elements 1 x 8 cm. The authors report that it delivers a CT optimal stimulus at 6cm/s, analogous to brushing at CT optimal velocities. However, it is far from clear that this device stimulates CT afferents optimally. Wessberg et. al. (2003) report CTs as having a mean receptive field size of 14.7 +/- 10.5 mm sq., so any given CT afferent is likely only stimulated by one S-CAT element. The S-CAT produces apparent motion rather than real motion, and the velocity of the stimulation is derived from the timing between successive activations of pneumatic elements. This means that the individual CTs are not going to be sensitive to the lateral velocity produced by this device.

Wessberg et. al. (2003) and others also showed that CTs respond to indentation. It is likely that the S-CAT does stimulate CTs, but how optimal it is cannot depend on its lateral velocity. I don’t think this issue should worry the authors too much, since it is clearly a pleasant stimulus that is comparable to soft brushing in many ways. However, it is a critical theoretical point that needs to be addressed. It is important to re-frame the nature of this stimulus, perhaps describing it as a “pleasant” or “affective” touch stimulus, rather than CT optimal.

J. Wessberg, H. Olausson, K. W. Fernström, and Å. B. Vallbo, ‘Receptive Field Properties of Unmyelinated Tactile Afferents in the Human Skin’, Journal of Neurophysiology, vol. 89, no. 3, pp. 1567–1575, Mar. 2003, doi: 10.1152/jn.00256.2002.

**Response to Reviewer’s # Major Comment 1:** We thank the reviewer for raising this important point, allowing us to better explain why we originally stated that the touch produced by the S-CAT can be described as CT-optimal touch, and how we have taken their point into consideration and amended our revised manuscript accordingly. Specifically, in a separate sample we had tested the effects of the S-CAT prototype we used in Experiment 2 and compared participants’ pleasantness ratings following touch via the S-CAT to those following manual and brush-mediated touch (Zheng et al., 2021). In that sample, we found that the S-CAT simulated the pleasantness effects of CT-optimal affective touch (Zheng et al., 2021). In other words, participants reported increased pleasantness at CT-optimal vs. suboptimal velocities, irrespective of touch medium (i.e., touch delivered via the S-CAT, via human touch, or via brush strokes). Given that we had examined the effect of touch medium on pleasantness ratings in that previous sample, examining the pleasantness ratings of participants following touch at CT-optimal vs. suboptimal velocities (delivered via the S-CAT vs. via brush strokes) was not a primary aim of our present Experiment 2. In hindsight, however, we agree with the reviewer that it is not possible to know if touch delivered by the S-CAT specifically targets the activation of CT-afferents. At the absence of evidence at the level of microneurography or equivalent, which would allow us to confirm the degree to which, or if to any degree at all, the S-CAT activates CT-afferents, we have reframed our S-CAT stimulus to ‘affective touch’. However, we had obtained behavioural data on baseline pleasantness ratings following touch at CT-optimal vs. suboptimal velocities, delivered via brush strokes and via the S-CAT, and consistent with the findings of Zheng et al. (2021) we also found that participants reported significantly higher pleasantness ratings following touch at CT-optimal vs. suboptimal velocities, irrespective of touch medium. We have now added this analysis in the Supplementary Material. Based on the above, we have now amended the following segments of our manuscript:

References:

1. Zheng, C. Y., Wang, K. J., Wairagkar, M., Von Mohr, M., Lintunen, E., & Fotopoulou, K. (2021, July). Comparing soft robotic affective touch to human and brush affective touch. In *2021 IEEE World Haptics Conference (WHC)* (pp. 352-352). IEEE.
2. Kirsch, L.P, von Mohr, M., Koukoutsakis, A., & Fotopoulou, A. (in prep) Vicarious touch: a potential substitute for social touch during touch deprivation.

In the Introduction we have amended line 151 – 161 (pp. 6-7) to read:

In a follow-up, preregistered, confirmatory study (Experiment 2) we used a similar setup but delivered affective ‘stroking touch’ with a validated, soft robotic sleeve (S-CAT; (77)), which elicits affective touch at CT optimal speeds (see Methods for details on the properties of the S-CAT). We investigated whether combining the above affective tactile emoticons with standard visual emotions would lead to greater perception of social approval and support than visual emoticons alone.

In the Main Aims, Design and Data Analysis section we have amended lines 515-519 (pp. 19) to read:

Finally, we controlled for the pleasantness elicited following affective touch delivered by the S-CAT vs. manual brush strokes to ensure that the S-CAT can elicit pleasantness ratings comparable to brush-stroke touch when delivered at CT-optimal velocities. The details and results of this analysis are reported in the Supplementary Material.

And, we have also amended lines 515-519 (pp. 19) to read:

The visual component was identical between the conditions but the tactile component of the visuotactile feedback was delivered via a wearable sleeve which can deliver affective touch at CT-optimal speeds (6cm/sec) and whose properties and effects have been described and validated in a separate study (S-CAT; (77); see details below).

We also added more details on the properties of S-CAT in the ‘Tactile feedback equipment and set-up’ section; lines 657 – 711 (pp. 24-25) now read:

The device wraps around the participant’s forearm (Fig 4 below), and the actuator design (with dimensions of 9cm x 4cm) which is covered with silicon on the inside is where participants feel the touch coming from. Specifically, the S-CAT has an array of pneumatic actuators which are arranged in a way to minimise spacing and is programmed to produce a tracing effect to simulate a caress-like touch gesture when actuated in a sequence (79). In other words, the eight air cells of the device (with dimensions of 1cm x 5.5cm each) are controlled individually by digital valves and a rippling effect which resembles that of skin stroking is created when inflating adjacent air cells with an overlapping time (77). Furthermore, a recent study looking at the properties of the S-CAT found that subjective pleasantness ratings were higher following slow, caress-like affective touch at CT-optimal velocities, as opposed to fast, CT-suboptimal touch, irrespective of stimulation type (i.e., skin-to-skin, brush stroking, or S-CAT touch; 77). Therefore, this prototype produces psychophysiological effects which can be compared to the CT-optimal affective touch which can be delivered either by a soft brush (like in Experiment 1) or skin-to-skin affective touch (79) when looking at the behavioural data (e.g., 77; see also Supplementary Material). At the absence, however, of microneurography studies to confirm this and the fact that the S-CAT is still at a prototype level, thus warranting further examination of its ability to optimally stimulate CT-afferents, we refer to the touch delivered by the S-CAT as ‘affective touch’. The temperature of the sleeve was kept at 36°C to keep it as close to human body temperatures as possible and reduce potential effects of low temperatures on social intent (89).

We also added the below in our Supplementary Materials; Lines 227 – 232 (pp. 10) now read:

For control purposes, we also collected baseline ratings of pleasantness (on a scale of 0 (*not at all pleasant*) to 100 (*extremely pleasant*) following touch delivered by the S-CAT and via brush strokes at CT-optimal (6cm/sec) and CT-suboptimal speeds (36cm/sec). We then ran an MLM with touch medium (manual touch via brush strokes vs. S-CAT) and touch velocity (optimal vs. suboptimal CT-touch) as interacting IVs. We examined the main effect and interaction of these IVs on participants’ pleasantness ratings. Participant ID was added as a random effect.

And, lines 287 – 300 (pp. 12) now read:

**Control Analysis: Pleasantness ratings were predicted by touch velocity but not by touch medium.**

We examined the main effect of touch medium and touch velocity and the interaction of these two variables on participants’ pleasantness ratings. We found a significant main effect of touch velocity, in that touch delivered at CT-optimal speeds, resulted in higher pleasantness ratings than touch delivered at CT-suboptimal speeds (*β*=20.78, SE=3.03, *t*(6.85), 95% CI=14.83 – 26.73, *p*<0.001, ICC=0.43, R_marginal_^2^=0.194, R_conditional_^2^=0.539). However, the effect of touch medium on pleasantness ratings was not significant (*β*=-2.81, SE=3.05, *t*(3.05), 95% CI=-8.78 – 3.16, *p*=0.356, ICC=0.43, R_marginal_^2^=0.194, R_conditional_^2^=0.539). The, interaction between these two IVs was also not significant (*β*=-3.36, SE=4.29, *t*(-0.78), 95% CI=-11.76 – 5.04, *p*=0.433, ICC=0.43, R_marginal_^2^=0.194, R_conditional_^2^=0.539). This result suggests that participants reported significantly higher pleasantness ratings following touch delivered at CT-optimal speeds (6cm/sec) compared to touch delivered at CT-suboptimal speeds (36cm/sec), irrespective of how the touch was delivered (touch medium).

**Reviewer #1, Major Comment 2:** Expt1 design: The selection of emoticons for high vs. low support needs to be better motivated. It’s not intuitive to me that thumbs up is high support and blue heart is low support (and why is it called “baseline blue heart”?). How did the authors make the choice? For both tactile and visual feedback, why was habituation not a concern for low support feedback, where only one stimulus was used?

**Response to Reviewer’s # Major Comment 2:** We thank the reviewer for their comment, allowing us to better clarify the rationale behind the emoticon selection and habituation concerns. When designing our first experiment, we first looked at how different emojis were used on popular social media platforms. For example, on X (formerly known as twitter), the icons (such as users have to touch on to “like” a tweet/post or to “share” a post) are outlined with a black line and only turn red once the social media user “likes” a tweet/post. On Facebook (or, Meta), the thumbs up outline was further developed to include more than one “message”/“post react” , i.e., a blue circle with a white thumbs up is used to “like” a post, while a red circle with a white heart is used to “love” (and they now also have emojis to communicate “care”, “laughter”, “surprise”, “sadness”, and “anger”), and therefore a “like” could be perceived as less supportive/engaging/strong than a “love’ circle. Our experimental design choices were based on these social media examples; in line with these examples, a “baseline blue heart” (at a lighter blue colour to the “like” on Facebook) could be perceived as less supportive/engaging/strong than a red heart or a thumbs up. We had also initially termed this heart as “baseline blue heart” to refer to the emoji’s design features before a user “clicks” on it, but we have now changed this term to “neutral blue heart” and explained why it is associated with low approval and support. Using the insights from Experiment 1 and qualitative feedback from participants (e.g., mixed feelings about a red heart following a post of negative valence, or sometimes confused about the meaning behind the neutral blue heart), in Experiment 2 we researched and chose different emoticons for the sentences of positive and negative valence, and emoticons with clearer meaning. We would also like to clarify the rationale of our tactile and visual emoticon choices and how we addressed habituation concerns. Specifically, our selection choice was informed by the fact that we primarily wanted to test optimal velocities of touch as emotional support touch and compare this ‘optimal’ tactile feedback to the effects of ‘optimal’ visual emoticons. This allowed us to differentiate among different velocities of touch, allowing us to have 3 velocities (and 3 visual emoticons) instead of 2 velocities (and 2 visual emoticons) in total. Having multiple velocities as well as two separate marked skin areas where touch was delivered allowed to address tactile feedback habituation concerns. Importantly, the feedback was not blocked, and all the different combinations of feedback (i.e., 3 tactile velocities, and 3 visual emoticons) were fully randomised within and between participants. We have made the following amendments to improve the readability and clarity of our revised manuscript:

The respective passage in the Introduction (lines 144-149; pp. 6) now reads:

Specifically, Experiment 1 simulated a social media communication platform during which healthy adults imagined they were reading their own typed posts (of either positive or negative emotional valence), while a confederate delivered tactile (using a brush) or visual feedback that communicated high support (e.g. stroking touch at CT-optimal velocities as a tactile emoticon or a red heart or thumbs up as a visual emoticon), or low support (e.g., stroking touch at CT-suboptimal velocities or a neutral blue heart as a visual emoticon).

Lines 193 – 196 (pp. 9) in the Methods now read:

Visual emoticons consisted of emoticon images with specific, socially supportive meanings (high support: red heart, or thumbs up), or less specific meanings (low support: neutral blue heart), expected to convey support less clearly.

We have now also added the below in the ‘Creation of affective visual feedback stimuli with visual emoticons’ section (lines 306 – 314; pp. 12):

To manipulate visual feedback, three widely used visual emoticons (high support: red heart, or thumbs up; or of less specific meaning, low support: neutral blue heart) were displayed beneath the sentence posts, as shown in Fig 1. The high and low support emojis were chosen following the emojis social media users can typically use to engage with a post (e.g., a red heart on X, and a blue thumbs up, or a red heart, among others to express “like” and “love”, respectively, on Facebook). To examine whether this separation of our visual emoticons did indeed affect participants’ perceived social intent, feedback support level was added as a IV in our main analyses.

We also added the below to lines 370 – 372 (pp. 13):

Overall habituation concerns were also addressed and minimised by fully randomising the (visual and tactile) feedback order within and between participants.

**Reviewer #1, Major Comment 3 and Minor Comment A:**

Authors’ note: Kindly note that we respond to these two comments jointly below.

**Major Comment 3:** Figure 1: It is certainly useful to have a diagram to explain the fairly complex design, but I did not find this diagram helpful. I noticed the following issues:

- “Valence” and “Feedback mode” are both variable names, but are represented differently, one embedded in the flow chart, the other as an annotation on the side.

- Valence being “positive” or “negative” doesn’t explain anything. Without reading the text, I would wonder what is it the valence of? Some example sentences would be nice here.
- In the text it says there were 8 conditions, but in the diagram it looks like there are four.

-Feedback support level is not represented in the diagram.

- The caption could give more details.

**Minor Comment A:**

Figures 2 and 4 – I’m not sure the content here justifies a whole figure. The visualisation of social media posts could perhaps be incorporated into Figure 1. Alternatively, Figures 2 and 4 could be combined.

**Response to Reviewer’s # Major Comment 3, and Minor Comment A:** We thank their reviewer for suggesting ways to improve the legibility of our Figure. Further to their major comment 3, in the minor comments they suggested to incorporate the visualisation of the social media posts (from Figures 2 and 4) into Figure 1, or to combine Figures 2 and 4. Following these two comments, we have decided to replace Figures 1, 2, and 4 with 2 new Figures: Figure 1 now shows the design of Experiment 1 and a visual example of the social media posts that were used, and we have created a separate figure which follows the same principles but for Experiment 2 (Figure 3). Following these two changes the current revision has updated figure numbers (and legends, where appropriate). For example, where we previously referred to Fig 3, we have now replaced it with Fig 2; we have also checked that these changes are consistent and correct throughout the manuscript).

We have now recreated the figure and tailored the original caption as appropriate, as shown below. Similarly, we have also created a new Figure for Experiment 2 (and respective caption), as shown below as well.

The amended figure for Experiment 1 can be seen below and the caption can be seen below and in the revised manuscript in lines 211-243 (pp. 9-10).

**Fig 1. A visual illustration of the experimental design and posts of Experiment 1.**

In Experiment 1 there were three main measures: Valence (i.e., the Valence of the posts’ context: Positive vs. Negative), Feedback Mode (Visual vs. Tactile emoticons) and Feedback Support Level (High vs. Low support). Specifically, in the positive valence half of the posts were paired with predetermined Visual feedback and the other half were paired with predetermined Tactile Feedback. The same was repeated for the posts with a negative valence. Further, within each Feedback Mode, the feedback participants received on half of the posts was of high Feedback Support Level (in the Visual feedback mode: red heart, and thumbs up emoticon; and, in the Tactile Feedback Mode: touch at 3cm/sec, and 6cm/sec), while in the other half they received low Feedback Support Level (in the Visual Feedback Mode: neutral blue heart; in the Tactile Feedback Mode: touch at 0.3cm/sec). This design led to 8 possible combinations and 8 posts per combination (i.e., 64 posts in total). The two post examples are examples of the post format used in the main task, showing what participants saw when getting feedback from the confederate. Participants were told in the beginning of the experiment that ‘TheTalSunX’ would be their username and ‘@_the_daily_tal_sun_x’ was the respective username of the platform from which they would be posting and getting Visual or Tactile feedback from. Every post displayed the username and handle on the top, as well as a profile picture (unbeknownst to them, the same for all participants) on the top left corner of the post, which consisted of a neutral image (bright red and orange dots). The timestamp on the top right corner of the post indicated how long ago the participant had posted on their profile (here, 58 minutes ago; all posts across all participants had the same timestamp). Below these standardised features all participants could see the sentence they posted (first, without the feedback, and then with the feedback). The example at the top right corner of the figure is a post of individualistic context, and positive valence (“I was upgraded to first class”) which received visual feedback from the confederate (here, high support as indicated by the ‘red heart’ emoticon). The example at the bottom right corner of the figure is a post of individualistic context and negative valence, which then received tactile feedback from the confederate (here, low support via brush strokes at 0.3cm/sec). The icons below the sentence would only appear when the participant was receiving visual feedback, and they are visually similar (although inactive here) to what users of actual social media platforms typically see when interacting which each other. The features, starting from the left are ‘reply’, ‘reshare’, ‘react’, ‘more’, the latter offering more functionalities.

The new figure for Experiment 2 can be seen below and the caption can be seen below and in the revised manuscript in lines 547-580 (pp. 21-22).

**Fig 3. A visual illustration of the experimental design and posts of Experiment 2.**

In Experiment 2 there were two main measures: Valence (Positive vs. Negative) and Feedback Mode (Visual vs. Visuotactile emoticons). Participants received visual and visuotactile feedback on posts of positive and negative valence. There were 4 possible combinations (e.g., “Valence: Positive, Feedback Mode: Visuotactile Emoticons) and 8 posts per combination (i.e., 48 posts in total; see below). and within each Valence and Feedback Mode combination the number of and type of visual emoticon reacts was varied (low, medium, and high; see below). However, for additional variability within each of the 4 combinations we varied the type of visual emoticon react (2 per intensity: low, medium, high; see below), thus resulting in 2 posts per Valence, Feedback Mode and Emoticon Reach combination. We also varied the number of emoticon reacts (low, medium, and high; see below). The number and type of emoticon reacts were not taken into consideration in the Experiment 2 analyses. The post examples are examples of what a participant would see when logged onto their ‘FaceJournal’ profile and receiving visual feedback. The screen display was similar to that of actual social media platforms on a browser (i.e., with the name of the website and the link on the top left, as well as typical browser functionalities on the top right (e.g., ‘bookmark’). Then, each post was individually shown like in this example. On each trial, participants’ name of choice (like the ‘Jane Doe’ example shown here) and the given icon they had set for their profile (always a round abstract image as the one shown here) were shown at the top left corner of the post. Below their name, participants could see how many minutes ago they posted on their profile (here, 2 minutes ago; all posts across all participants had the same timestamp), with the ‘connect’ logo of the platform next to the timestamp (as indexed by the grey earth-like icon). In the example at the top right corner of the figure, a post of positive valence is shown and thus the visual feedback is a smiley visual emoticon (i.e., a low intensity visual emoticon) with seemingly 24 such reactions (i.e., a number of likes from the low range). To create a more realistic experience of FaceJournal interactions, below the other users’ reactions participants could also see the ‘React’ and ‘Comment’ functionalities social media users also have below their own posts (should they wish to also react on their post and/or comment). The example at the bottom right corner of the figure is a post with the same design features as the top post, but with a sentence of negative valence and thus the emoticon react is a shocked face (here, medium intensity). The specific post was also paired with predetermined visuotactile feedback, thus the participant also received affective touch via the S-CAT at the same time as the visual emoticon appeared on their screen.

**Reviewer #1, Major Comment 4, and Minor Comment B:**

Authors’ note: Kindly note that we respond to these two comments jointly below.

**Reviewer #1, Major Comment 4:**

Figures 3 and 7: the caption should describe all the elements of the figure and say what they mean. What does each dot represent, is it a single rating, or an average per participant? What are the confidence intervals, and the point in the middle? Is it mean +/- SD, or something else? EMM and 95% CI? How do these figures relate to the statistical models?

**Reviewer # 1 Minor Comment B:**

Figures 3 and 7 – It is difficult to compare these figures with the valence and feedback format being swapped (colour vs. x-axis). The error bars are very difficult to see, the authors could consider making them more visible in some way, e.g. making the dots partially transparent or paler. The distributions should also be partially transparent since they are overlapping.

**Response to Reviewer’s # Major Comment 4 and Minor Comment B:** Figures 3 and 7 (now changed to Figures 2 and 6 after the changes made to the initial Figures 1, 3, and 4; please see response to Reviewer’s #1 Major Comment 3) have been reproduced to improve the clarity of the error bars and distributions. For Experiment 1 we created a second figure (Fig. 2, Panel B) to allow comparison of social intent scores across the two experiments when feedback is of high support only. We have also added more information in the caption.

Lines 455 – 467 (pp. 16 – 17) now read:

**Fig 2. Social intent scores following visual and tactile feedback.** Panel A shows participants’ social intent scores following feedback at high and low support levels. Panel B shows participants’ social intent scores following feedback (high support only) on positive and negative posts. The rainclouds represent the distribution of Social Intent when grouped by Feedback Mode and Support Level (in Panel A) and when grouped by Feedback Mode and Valence (in Panel B). The dots represent the average score per participant for each combination of Feedback Mode and Support Level (in Panel A) and Feedback Mode and Valence (in Panel B). Error bars indicate the 95% confidence interval (CI) around the mean (Mean of Social Intent +/- 95% CI). The dot on the error bar is the sample mean of Social Intent for the respective combination of Feedback Mode and Support Level (in Panel A) and Feedback Mode and Valence (in Panel B). The horizontal dashed line (at y = 0) indicates that the Social Intent scores could not be below 0.

And, lines 815 – 823 (pp. 29) now read:

**Fig 6. Social intent scores following visual and visuotactile feedback.** Participants’ social intent scores following visual and visuotactile feedback on positive and negative posts. The rainclouds represent the distribution of Perceived Social Intent grouped by Feedback Mode and Valence. The dots represent the average score per participant for each combination of Feedback Mode and Valence. Error bars indicate the 95% confidence interval (CI) around the mean (Mean of Perceived Social Intent +/- 95% CI). The dot on the error bar is the sample mean of Perceived Social Intent for the respective combination of Feedback Mode and Valence. The horizontal dashed line (at y = 0) indicates that the Perceived Social Intent scores could not be below 0.

**Reviewer #1, Major Comment 5:** The authors have clearly put a lot of effort into careful consideration of statistical models, power analysis, and pre-registering experiment 2, and I commend them for that. Further, I commend the authors on providing clearly labelled data and reproducible analyses. I did not do a thorough data/code review, but performed a few spot checks, and did not find any issues.
**Response to Reviewer’s # Major Comment 5:** We thank the reviewer for their positive comment. We would also like to note that we have now uploaded an updated R script on our github repository with the additional analyses and graphs we included in the current revision.

**Reviewer #1, Major Comment 6:** It is strange to me that the SCR and HR results were not included in the main manuscript. It would have been nice to see a figure showing these data. Even though there was no significant main effect, I think the results are of sufficient interest to be included.

**Response to Reviewer’s # Major Comment 6:** Given that the manuscript presents two experiments with a number of hypotheses and related analyses, we believe it is important to keep it clear and precise. At the same time, we do agree with the reviewer that the physiological results are interesting to include in the main manuscript. We have addressed this by adding a summary of the key findings and figures to show the physiological data and cross-reference the Supplementary Material where full details on the results are provided.

We have now added the below in lines 509 – 511 (pp. 19):

We also measured the effects of feedback mode on physiological measures, such as heart rate, and skin conductance rate. These physiological and exploratory analyses were secondary aims of the present paper and the results are briefly reported below and reported in detail in the Supplementary Material.

In the Data Analysis section (lines 611 – 618; pp. 23) we added:

For the fourth hypothesis, we examined the effect of feedback mode on three physiological measures, namely HR, SCR, and heart rate variability (HRV). We ran an MLM with baseline corrected HR, SCR, and HRV scores as our separate DVS, feedback mode as the IV and participant ID and block as random effects. We then examined the interaction between feedback mode and traits (rejection sensitivity and attachment anxiety). Details on physiological measure calculations are presented in the Supplementary Material as the examination of feedback mode on physiological measures was a secondary aim of Experiment 2.

In the Results section (lines 881 – 912; pp. 31 – 32) we added:

**Hypothesis 4: Visuotactile feedback did not lead to greater regulation of physiological measures in comparison to Visual feedback.**

Contrary to our hypotheses, visuotactile feedback did not regulate physiological measures (namely, heart rate (HR), skin conductance rate (SCR), and heart rate variability (HRV); Fig 7) more than visual feedback (HR: *β*=-0.15, SE=0.33, *t*(-0.47), 95% CI=-0.79-0.48, *p*=0.638, ICC=0.91, R_marginal_^2^=0.000, R_conditional_^2^=0.910; SCR: *β*=-0.02, SE=0.03, *t*(-0.91), 95% CI=-0.07-0.03, *p*=0.365, ICC=0.89, R_marginal_^2^=0.001, R_conditional_^2^=0.887; HRV: *β*=0.0008, SE=0.001, *t*(0.769), 95% CI=-0.0012-0.0027, *p*=0.442, ICC=0.93, R_marginal_^2^=0.000, R_conditional_^2^=0.927). Interestingly, in preregistered analyses examining the role of rejection sensitivity and attachment anxiety we found that visuotactile feedback led to more downregulation of SCR in individuals with high rejection sensitivity scores in comparison to visual feedback (*β*=0.01, SE=0.01, *t*(2.12), 95% CI=0.00-0.02, *p*=0.034, ICC=0.89, R_marginal_^2^=0.019, R_conditional_^2^=0.896). A similar pattern was observed in HRV – both across post valence, and within each valence separately): the regulation in HRV was better explained by the visuotactile feedback in comparison to the visual feedback in individuals with higher trait anxiety scores (Across valences: (*β*=0.0005, SE=0.0002, *t*(3.254), 95% CI=0.0002-0.008, *p*=0.001, ICC=0.93, R_marginal_^2^=0.064, R_conditional_^2^=0.933); Negative Valence: (*β*=0.0005, SE=0.0002, *t*(2.082), 95% CI=0.0000-0.001, *p*=0.037, ICC=0.92, R_marginal_^2^=0.065, R_conditional_^2^=0.921); for full details see Supplementary Material).

**Fig 7. Changes in physiological measures following visual and visuotactile feedback.** Heart rate (HR; Panel A) and skin conductance rate (SCR; Panel B) were baseline corrected – in other words, the mean HR and SCR scores from each condition block (e.g., visuotactile feedback on negative posts) were subtracted from the average HR and SCR scores we obtained before the task. Heart Rate Variability (HRV; Panel C) was calculated as the time elapsed between successive inter-beat intervals. The y’y axis on Panel C represents the mean of HRV values obtained during the main task, when participants were receiving feedback on their posts. The rainclouds – in all three panels – represent the distribution of the physiological measure grouped by Feedback Mode and Valence. The dots represent the average score per participant for each combination of Feedback Mode and Valence. Error bars indicate the 95% confidence interval (CI) around the mean (Mean of Baseline Corrected HR in Panel A, Baseline Corrected SCR in Panel B, and HRV in Panel C).

**Reviewer # 1 Minor Comments:**

**Reviewer #1, Minor Comment 1:** The authors have made a thorough review of the relevant literature, which is commendable. I wonder if it is necessary to cite so many review articles, given that in most cases the authors already provide several citations to original research articles.

**Response to Reviewer’s #1 Minor Comment 1:** We have included both original research articles and review articles given that the two experiments are rather novel (in that no previous study, to our knowledge, has looked at the effects of affective touch on social media communications). We believe it is important that the background and rationale provide a detailed and thorough description of what is currently known, and cite papers which not only offer insights to original research articles but also to papers which offer a more collective review of the existing literature in order to introduce our rationale behind the aims, hypotheses, and design of our two experiments.

**Reviewer #1, Minor Comment 2:** There are quite a lot of minor typos/spelling/grammatical errors. I started pointing them out, but there were enough that I feel it was getting out of scope for my role as reviewer. I suggest the authors do another editing pass to iron it out.

**Response to Reviewer’s #1 Minor Comment 2:** We have proofread the manuscript and Supplementary Material and fixed all typos, spelling, and grammatical errors.

**Reviewer #1, Minor Comment 3:** The IV is sometimes described as “perceived social support” and at other times as “perceived social intent”, and I suggest that a single consistent description should be used. I think the former is more intuitive, especially when reporting results e.g. “the participants perceived greater social support …” reads better than “the participants perceived greater social intent …”

**Response to Reviewer’s #1 Minor Comment 3:** Thank you – we have checked the manuscript to ensure “perceived social intent” is used consistently. Although “perceived social support” reads better, it does not include the all the emotions that the confederate/user communicated to the social media poster/participant (i.e., social approval and support in Experiment 1, and social approval, validation, support, and sympathy in Experiment 2). We have used the term “perceived social intent” to refer to the different intentions the user wanted to communicate to the participant.

**Reviewer #1, Minor Comment 4:** 50-55, sentence is very long

**Response to Reviewer’s #1 Minor Comment 4:** we have split the sentence. The sentences now read (lines 53 – 59; pp. 3):

Social media are a motivator for information sharing, fulfilment of social connection, and relationship maintenance (e.g., (1–3)). However, intense social media consumption (e.g., endless platform scrolling) may also hinder social connections, induce phubbing (the habit of favouring a mobile device over a physically present person) and negatively influence individual wellbeing (e.g., (4–8)); for reviews on social media, (dis)connection and mental health see (9,10)).

**Reviewer #1, Minor Comment 5:** 90, not clear what is meant by ”experience” here

**Response to Reviewer’s #1 Minor Comment 5:** Experience here refers to pain experience. (Pain during pain anticipation and experience). We have edited lines 97 – 100 (pp. 4) to read:

This social touch, and particularly CT touch, reduces negative physiological and affective states such as the pain of social rejection ((15); for review see (27,33)), physical and social pain (34–41), stress (for reviews see (25,42)), pain during pain anticipation (43) and pain experience (15,39–41,44).

**Reviewer #1, Minor Comment 6:** 136-47: somewhere here, briefly describe how the touch is delivered in Experiment 1 (brush)

**Response to Reviewer’s #1 Minor Comment 6:** We have clarified in the sentence (lines 144 – 149; pp. 6) which now reads:

Specifically, Experiment 1 simulated a social media communication platform during which healthy adults imagined they were reading their own typed posts (of either positive or negative emotional valence), while a confederate delivered tactile (using a brush) or visual feedback that communicated high support (e.g. stroking touch at CT-optimal velocities as a tactile emoticon or a red heart as a visual emoticon), or low support (e.g., stroking touch at CT-suboptimal velocities or a neutral blue heart as a visual emoticon).

**Reviewer #1, Minor Comment 7:** 138 “emtional” should be “emotional”

**Response to Reviewer’s #1 Minor Comment 7:** We have fixed this as well as all other typos in the manuscript.

**Reviewer #1, Minor Comment 8:** 195 what does “pre-determined” mean here? Was the visuotactile feedback in expt 2 not pre-determined?

**Response to Reviewer’s #1 Minor Comment 8:** We thank the reviewer for this question, but we have now edited out this section (also in line with their feedback to improve Figure 1; please see Reviewer #1, Major Comment 3, and Minor Comment A) and hence this comment is no longer applicable.

**Reviewer #1, Minor Comment 9:** 202 “resulting in 8 posts were combination” something wrong with this sentence

**Response to Reviewer’s #1 Minor Comment 9:** We thank the reviewer for bringing our attention to this phrasing mistake, but we have now edited out this section (also in line with their feedback to improve Figure 1; please see Major Comment 3) and hence this comment is no longer applicable.

**Reviewer #1, Minor Comment 10:** 203-207 why is expt 2 mentioned here when it has its own section later?

**Response to Reviewer’s #1 Minor Comment 10:** Experiment 2 was mentioned in the caption of Figure 1, as the Figure initially presented the design of both Experiments. However, in the current revision, and also following their suggestion (please see Reviewer #1 Major Comment 3, and Minor Comment A), we have now replaced Figures 1, 2, and 4 with new Figures (Fig 1 and Fig 3) and hence this comment is no longer applicable.

**Reviewer #1, Minor Comment 11:** 482-485 sentence is long and unwieldy

**Response to Reviewer’s #1 Minor Comment 11:** We have improved the legibility and clarity of this sentence by splitting it into two shorter sentences and rephrasing previously unclear terms. The sentence in lines 625 – 630 (pp. 23) now reads:

In preregistered Experiment 2 we examined whether visuotactile feedback (i.e., tactile feedback in addition to visual feedback) would be associated with increased levels of perceived social intent as opposed to visual feedback only. Contrary to Experiment 1, in Experiment 2 we did not explore feedback optimality differences.

**Reviewer #1, Minor Comment 12:** Expt 2 –it is not clear where the participant thinks the touch comes from, do they believe the experimenter chooses when it is delivered, that it is based on emojis/likes etc., or that it is a feedback option that some/all of the “other participants” (confederates) are selecting in addition to emojis?

**Response to Reviewer’s #1 Minor Comment 12:** We apologise for the confusion, and we have now edited the entire section to explain that the feedback was sent from the ‘other’ users participants met at the beginning of the session in another room. We explain that the users can send visual (emojis and likes) feedback or visuotactile feedback (visual feedback in addition to touch via the wearable sleeve) to the participant in real time. In reality, and unbeknownst to the participant (until the debriefing at the end), there was no “other participant” who chose when and what type of feedback to send to the participant. Instead, the feedback was predetermined. We have edited lines 725 – 738 (pp. 26) to clarify this:

Upon arrival, participants met other FaceJournal users who were introduced to them in another room at the onset of the experiment. Participants were told that the other users would be logged into the platform at the same time from that room. Participants were also told that the ‘other’ users would be able to read the participant’s posts that the participant would be writing and posting on their profile in real time, and deciding – also in real time- whether to send them visual, or visuotactile feedback and a number of ‘likes’. The participants were told that the visual feedback would be in the form of visual emoticons, and if the “other” users wanted to send tactile feedback as well, they would do that by sending touch via a wearable sleeve (i.e., S-CAT) that the participant would be wearing during the experiment. In reality, the ‘other’ users (unbeknownst to the participants until the debriefing at the end of the session) were lab members of the research team and they were not involved in the feedback participants received. Instead, the visual and visuotactile feedback the participants would be receiving during the experiment was all predetermined and computer generated. Participants were told they would not be meeting the other users again at any point during the experiment.

**Reviewer #1, Minor Comment 13:** 644-645 – Alpha was set at 0.05, so it is inappropriate to say “We found a tendency towards significance”.

**Response to Reviewer’s #1 Minor Comment 13:** We have corrected this. Lines 857 – 861 (pp. 30) now read:

We found a non-significant trend on the effect of feelings of Safety on perceived social intent (*β*=0.22, SE=0.12, *t*(1.87), 95% CI=-0.01-0.46, *p*=0.061, ICC=0.76, R_marginal_^2^=0.050, R_conditional_^2^=0.722), but no significant interaction between Safety and valence (*β*=-0.14, SE=0.09, *t*(-1.52), 95% CI=-0.33-0.04, *p*=0.127, ICC=0.73, R_marginal_^2^=0.119, R_conditional_^2^=0.764).

**Reviewer #1, Minor Comment 14:** I tried to run the manuscript through <http://statcheck.io/>, however it didn’t seem to detect most of the tests because of the formatting of the pdf. I recommend the authors use this tool to check for issues in their statistical reporting, e.g. copy/paste errors.

**Response to Reviewer’s #1 Minor Comment 14:** Thank you for this suggestion. We tried using the tool but encountered the same issue as the reviewer, with the software not recognising some of our tests. We have however gone back and re-run our analyses and double-checked that no typos or mistakes were reported in the original submission.

**Reviewer #2:**

This is an interesting, preregistered (exp. 2 only), lab-based study which aims to examine the role of touch in social media communications by comparing the effects of visuo-tactile emoticons, delivered according to CT-optimal and CT-suboptimal properties of affective touch as compared to standard, visual-only emoticons. Authors report that irrespective of whether participants received tactile feedback delivered by brush stroking by a confederate (Exp. 1), or remotely via a wearable sleeve (Exp. 2), tactile and visuo-tactile emoticons appear to overall communicate prosocial intent more clearly, particularly during communications of positively valenced content. The study is very well designed and executed. It also considers interesting individual traits measured by means of several interesting self-reports, which my moderate the effects obtained. I particularly liked the idea of exploring physiological responses associated to (touch mediated) social media communications, despite the negative results obtained according to which HR, HRV and SCR were not downregulated more in the visuo-tactile feedback mode as opposed to the visual feedback mode. Limitations are fairly acknowledged and discussed in the Discussion section. Overall, I strongly support the publication of this study in PLoS One, once, my only minor comments are acknowledged:

**Reviewer #2, Minor Comment 1:** Page 10, Line 223: please check not relevant information reported here (date, time?);

**Response to Reviewer’s #2, Minor Comment 1:** The sentence in lines 221-224 describes the emotions that accompanied our constructed sentences and does not include any irrelevant information (such as the date and time the sentence was ‘posted’ on the social media platform we created for experimental purposes) and we believe the reviewer is probably referring to the caption of Figure 2 which describes all the features of our post exemplar (lines 248 – 267 on the original manuscript). However, Figures 1 and 2 have now been combined (now Fig 1 in the revised manuscript) and we have edited to clarify that the timestamp was the same across all posts that the participants viewed (the date of the post was not shown in any of our posts). Please see detailed caption below our response to reviewer’s #1 Major Comment 3 (pp. 7-8 here).

**Reviewer #2, Minor Comment 2**: Page 20, Line 479: please clarify what you mean by ‘enhanced’ visual feedback;

Response to Reviewer’s #2, Minor Comment 2: We apologise for the confusion and would like to note that also following Reviewer’s #1 Minor Comment 11, we have now amended this sentence to improve the legibility (please see our response to reviewer #1 in page 15). Therefore, this comment is no longer applicable as we have now removed the phrase ‘enhanced visual feedback’ (which referred to the combination of tactile and visual feedback) and instead explained that visuotactile feedback is tactile feedback in addition to visual feedback.

**Reviewer #2, Minor Comment 3:** Page 22, Line 535: please double-check typo ‘penrumatic actuators’ should read pneumatic actuators;

**Response to Reviewer’s #2, Minor Comment 3:** Thank you for spotting this. We have now fixed it. Lines 711 – 714 (pp. 25) now read:

(A) Shows the inside materials of the S-CAT. This is the side that contacts with the skin, and the pneumatic actuators covered by the silicon layer on the outside (as shown in the image) were positioned on the participant’s forearm, as it is where touch was being delivered from (for more see (77)).

**Reviewer #2, Minor Comment 4:** Page 27: it would be good to see a brief summary of the results obtained by experiment 2.

**Response to Reviewer’s #2, Minor Comment 4:** We have added a brief summary. Lines 914 – 927 (pp. 32 – 33) now read:

**Summary**

In summary, participants reported greater levels of social intent following visuotactile feedback as opposed to visual feedback alone, and especially on posts of positive valence but not when looking at the effects of visuotactile vs. visual feedback on posts of negative valence. Moreover, the extent to which participants identified with (but not related to) the posts was predictive of greater levels of social intent, irrespective of feedback mode. As expected, increased feelings of safety and trust when wearing the S-CAT were predictive of a higher rating of social intent in the visuotactile feedback mode. Moreover, pleasantness ratings were higher following touch delivered at CT-optimal velocities irrespective of how touch was delivered (i.e., via the S-CAT or brush strokes; see Supplementary Material for details on this analysis). Finally, feedback mode was not predictive of physiological (HR, SCR, and HRV) changes, but we found that higher rejection sensitivity scores and higher trait anxiety scores explained more of the SCR and HRV variance, respectively following visuotactile feedback in comparison to visual feedback.

**Reviewer #2, Minor Comment 5:** Methods Experiment 1 (and 2): it would be good to see additional information about ethnicity of participants, and/or in general a more thorough description of the demographic of the two populations (also to allow for better comparison between the two populations of the two studies).

**Response to Reviewer’s #2, Minor Comment 5:** Thank you for the suggestion – we have now added more demographic information on the ethnicity and sexual orientation of our two samples. We have added Table 1 (in the Methods section of Experiment 1) and Table 2 (in the Methods section of Experiment 2) to present this information. This demographic information, across both samples, is consistent with the typical, UCL student population. The Tables and amended material are presented in lines 173 – 180, and 184 (Experiment 1; pp. 7-8), and in lines 476 – 478, and 485 (Experiment 2 pp. 17-18)

A total of N=39 participants with no prior history of psychiatric, neurological, and/or dermatological conditions were recruited from the University College London (UCL) research participant database (SONA; testing took place between 11 June 2019 and 30 September 2019), but two participants were removed from the dataset due to noncompliance with instructions and one due to software failure, respectively; final sample N=36 (50% male; *M_Age=_*25.08, *SD*_Age_=3.83; Table 1). The sample demographic information is consistent of the typical UCL student population.

| **Table 1. Demographics of Experiment 1 Participants** | | | |
| --- | --- | --- | --- |
|  |  | N | % |
| GENDER | Female | 18 | 50.00 |
|  | Male | 18 | 50.00 |
| SEXUAL ORIENTATION | Heterosexual | 28 | 77.78 |
|  | Homosexual | 3 | 8.33 |
|  | Bisexual | 2 | 5.56 |
|  | Other | 1 | 2.78 |
|  | Prefer not to say | 2 | 5.56 |
| ETHNICITY | White | 14 | 38.89 |
|  | Asian | 15 | 41.67 |
|  | Other* | 7 | 19.44 |
| Note. Other* refers to one of the following: Middle Eastern, African American, Latino, and Mixed Race. | | | |

The final sample consisted of N=52 participants (*M*_Age_=22.04, *SD*_Age_=4.44; N=37 female; Table 2) and as in Experiment 1, was also characteristic of the typical UCL student population.

| **Table 2. Demographics of Experiment 2 Participants** | | | |
| --- | --- | --- | --- |
|  |  | N | % |
| GENDER | Female | 38 | 73.08 |
|  | Male | 13 | 25.00 |
|  | Other | 1 | 1.92 |
| SEXUAL ORIENTATION | Heterosexual | 34 | 65.38 |
|  | Homosexual | 0 | 0.00 |
|  | Bisexual | 5 | 9.62 |
|  | Other | 9 | 17.31 |
|  | Not sure | 3 | 5.77 |
|  | Prefer not to say | 1 | 1.92 |
| ETHNICITY | White | 21 | 40.38 |
|  | Asian | 26 | 50.00 |
|  | Other | 5 | 9.62 |
| Note. Other* refers to one of the following Middle Eastern, African American, and Mixed Race. | | | |

**Reviewer #2, Minor Comment 6:** Discussion: Whilst I appreciate a direct comparison between perception of social intent between manual and mediated affective touch, given the different samples, designs and measurements of Experiments 1 and 2, cannot be drawn, yet, it would be useful to provide an explanation (or speculation) as to why overall, differences do not occur in the two touch media. I was actually surprised (apologies if I missed this!) by the fact that I do not seem to find a self-report evaluation of the preference (pleasantness) or intensity for touch induced manually as opposed to touch delivered by the soft robotic device for receiving feedback.
**Response to Reviewer’s #2 Minor Comment 6:** Thank you for this suggestion to help improve our manuscript. As regards to social intent, we cannot compare statistically between the two Experiments and it is therefore counter-indicated to draw conclusions. In consideration with the reviewer’s point and suggestion we have however added a line in our limitations section to explain why although we do not have the data to examine this explicitly, we speculate that participants across both Experiments may have reported increased social intent following tactile vs visual feedback because of comparable effects of brush and S-CAT mediated touch. Moreover, in Experiment 2 we found that increased feelings of safety and trust towards the S-CAT were predictive of increased social intent. This is in line with another recent study which showed that robotic touch effects can depend on the degree of perceived awkwardness of the robotic device (Kirsch et al., in prep). Please see amendments below. Additionally, we would also like to note that the while we have baseline ratings of pleasantness following S-CAT vs brush-mediated touch, the focus in Experiment 2 was on the social intent ratings and we had therefore not included this analysis in our manuscript (as we have a separate study examining the effects of touch medium on pleasantness; please see Zheng et al., 2021). However, in response to Reviewer’s #2 comment, as well as Reviewer’s #1, Major Comment #1, we have now included the analysis looking at the effect of touch medium and velocity on pleasantness ratings in the Supplementary Material. Please refer to our response to Reviewer’s #1, Major Comment #1 for details on this analysis and respective manuscript amendment.

We have amended the following passage in the discussion by adding the section highlighted in yellow below (lines 1058 – 1068; pp. 38).

A second limitation is that as regards to social intent scores across the two experiments, we cannot statistically compare to draw conclusions as to why overall participants from both experiments seemed to report increased scores of social intent following (visuo)tactile feedback as opposed to visual feedback only. We speculate that this similarity across the two experiments – at the absence of explicit, quantitative analyses – could be explained by the comparable effects and responses elicited by brush stroke and S-CAT mediated touch. Moreover, our finding that increased feelings of safety and trust towards the S-CAT (in Experiment 2) were predictive of higher social intent scores is in line with another recent study in which we showed that robotic touch effects (e.g., in eliciting calmness) can depend on the degree of perceived pleasantness and awkwardness of the robotic device (100).

**Reviewer #2, Minor Comment 7:** It would be good to see a bit more consideration of the secondary results obtained by using self-report scales which go beyond correlations with the TAS, particularly in relation to the STQ and BAS, and beyond whether individual differences in these traits can be linked to the amount of perceived social intent during the main task as reported in the supplemental analyses (page 29, Lines: 696-699).

**Response to Reviewer’s #2, Minor Comment 7:** We thank the reviewer for this comment. In Experiment 1 we found a negative correlation between social intent and BAQ and a positive correlation with social media use frequency and TAS (and no correlation with STQ). We have added a line for BAQ and STQ but would like to note that given that these analyses were secondary and correlational we cannot comment explicitly on the role of these individual differences on social intent. However, building upon these findings, in Experiment 2 we focussed on specific traits we expected to be associated with physiological changes following feedback during social media communication (e.g., trait anxiety and rejection sensitivity) and discuss these in lines 1038 – 1049 (pp.37 in the revised manuscript).

Lines 958 – 967 (pp. 35) now read:

Interestingly, perceived social intent was positively correlated both with actual social media engagement frequency and the ability to recognise and describe emotions (as measured using the Toronto Alexithymia Scale (TAS; (90)), and negatively correlated with how aware of and connected one is to what their body communicates (as measured using the Body Awareness Questionnaire; 91). Interestingly, we found no correlation between attitudes to social touch (as measured using the Social Touch Questionnaire; 92) and social intent scores. Thus, while we have indications that there can be individual differences in non-verbal emotional communication during social media, future studies with larger samples could further examine how exactly tactile or visuotactile emoticons could alter the habits and the effects of social media use.
